# Clinical Evaluation of the Efficacy and Tolerability of Rigenase^®^ and Polyhexanide (Fitostimoline^®^ Plus) vs. Hyaluronic Acid and Silver Sulfadiazine (Connettivina^®^ Bio Plus) for the Treatment of Acute Skin Wounds: A Randomized Trial

**DOI:** 10.3390/jcm11092518

**Published:** 2022-04-29

**Authors:** Raffaele Russo, Albino Carrizzo, Alfonso Barbato, Barbara Rosa Rasile, Paola Pentangelo, Alessandra Ceccaroni, Caterina Marra, Carmine Alfano, Luigi Losco

**Affiliations:** 1 Plastic Surgery Unit, Department of Medicine, Surgery and Dentistry, University of Salerno, Baronissi, 84081 Salerno, Italy; rafrusso@unisa.it (R.R.); acarrizzo@unisa.it (A.C.); barbara.rasile@gmail.com (B.R.R.); paolapentangelo1995@gmail.com (P.P.); aceccaroni@unisa.it (A.C.); camarra@unisa.it (C.M.); 2 Vascular Physiopathology Unit, IRCCS Neuromed, 86077 Pozzilli, Italy; 3 U.O.C. di Chirurgia Plastica Ricostruttiva, Azienda Ospedaliera Universitaria OO.RR. San Giovanni di Dio e Ruggi d’Aragona, Via S. Leonardo 1, 84131 Salerno, Italy; alfonso.barbato@sangiovannieruggi.it

**Keywords:** wound healing, acute skin wound, hyaluronic acid, aqueous extract of *Triticum vulgare*, TVE, Rigenase, Connettivina Bio Plus, Fitostimoline Plus, randomized trial

## Abstract

Objectives: Compare the efficacy and tolerability of Connettivina^®^ Bio Plus (Group A) gauze and cream, and Fitostimoline^®^ Plus (Group B) gauze and cream for the treatment of acute superficial skin lesions. Design: Single-center, parallel, randomized trial. A block randomization method was used. Setting: University of Salerno—AOU San Giovanni di Dio e Ruggi d’Aragona. Participants: Sixty patients were enrolled. All patients fulfilled the study requirements. Intervention: One application of the study drugs every 24 h, and a six-week observation period. Main outcome measures: Efficacy and tolerability of the study drugs. Results: In total, 60 patients (Group A, *n* = 30; Group B, *n* = 30) were randomized; mean age was 58.5 ± 15.8 years. All patients were included in the outcome analysis. Total wound healing was achieved in 17 patients undergoing treatment with Connettivina^®^ Bio Plus and 28 patients undergoing treatment with Fitostimoline^®^ Plus. The greater effectiveness of the latter was significant (*p* = 0.00104). In Group B, a significantly greater degree of effectiveness was observed in reducing the fibrin in the wound bed (*p* = 0.04746). Complications or unexpected events were not observed. Conclusions: Both Connettivina^®^ Bio Plus and Fitostimoline^®^ Plus are secure and effective for treating acute superficial skin lesions. Fitostimoline^®^ Plus was more effective than Connettivina^®^ Bio Plus in wound healing of acute superficial skin lesions, especially if fibrin had been observed in the wound bed.

## 1. Introduction

Nowadays, health care professionals are frequently called upon to manage acute or chronic wounds; their management may often lead to complications representing a “silent epidemic” [1]. A deep knowledge of the complex synchronized cascade involved in the anatomical and functional integrity of the skin is essential; on the other hand, methods and materials for wound management should be well acknowledged [2,3,4,5]. Timely treatment of acute skin lesions is paramount to prevent delayed wound healing, chronicization of the wound, and subsequent increases in health care costs [6].

Hyaluronic acid (HA) is a major component of the extracellular matrix (ECM) of the skin, joints, and many other tissues [7]. Owing to its remarkable biomedical and tissue regeneration potential, HA is widely employed in modern medicine under different formulations such as gauzes, fillers, injective, creams, and gels. It shows a wide range of pharmacological activities, including anti-inflammatory [8], wound-healing and tissue-regenerating [9,10,11], immunomodulatory [12], and cosmetic properties [13]. HA is involved in each phase of wound healing: it stimulates cell migration, differentiation, and proliferation; moreover, it regulates ECM organization and metabolism. HA was combined with silver sulfadiazine (SSD), which prevents colonization of the wound [14], to create an advanced dressing marketed under the brand name Connettivina^®^ Bio Plus gauzes and cream (Fidia Farmaceutici S.p.A., Abano Terme, Italy).

Rigenase^®^ is a specific extract of *Triticum vulgare* (TVE); it retains a scavenger effect against free radicals, thus showing significant antioxidant activity. It also maximizes the tissue regeneration process through an increase in chemotaxis, fibroblastic proliferation, and maturation. These properties are due to the increase in protein synthesis, proline uptake, and the upregulation of many fundamental factors such as MMP-2, MMP-9, collagen I, and elastin [15]. It is used to treat pressure sores, venous leg ulcers, wounds, burns, delays in scarring, dystrophic conditions, and, more generally, problems related to re-epithelialization or tissue regeneration [16]. Rigenase^®^ was combined with poliesanide (PHMB), which prevents colonization and contamination of the wound [17], to create the medical device Fitostimoline^®^ Plus gauzes and cream (Farmaceutici Damor S.p.A., Napoli, Italy).

Fitostimoline^®^ Plus and Connettivina^®^ Bio Plus are widely used, and they are both considered effective treatments of acute and chronic skin lesions; however, to the best of our knowledge, they have never been compared for the treatment of acute skin lesions. The aim of our study was to compare the efficacy and tolerability of two advanced dressings for the treatment of acute superficial skin lesions.

## 2. Patients and Methods

This was a single-center, equally randomized (1:1), parallel group study conducted at the University of Salerno, AOU San Giovanni di Dio e Ruggi d’Aragona, from September 2020 to December 2021. Eligible patients were all adults above 18 years of age and presenting with an acute skin lesion related to burn, trauma, or surgical wound dehiscence.

Patients with any of the following conditions were excluded from study participation: pregnancy or breastfeeding; inadequate contraceptive procedures in fertile women; chronic concomitant treatment with local antiseptics, use of anti-inflammatory (steroid and non-steroidal), analgesic, antineoplastic, or immunosuppressive drugs; non-therapeutic use of psychoactive substances; abuse of drugs and/or alcohol; immunodeficiencies (i.e., HIV infection); current neoplastic diseases; known allergies, hypersensitivity, or intolerance to any of the substances administered in this trial; any medical or non-medical condition which could significantly reduce the possibility of obtaining reliable data and achieve the objectives of the study; any condition that may affect the validity of the informed consent and/or compromise the patient’s adherence to the study procedures; treatment with any study dressings in the last 30 days prior to the start of the study; a previous enrollment in this study.

According to the results of a pilot study conducted in our University Hospital, healing was expected in 90% of patients treated with Fitostimoline^®^ Plus (Farmaceutici Damor S.p.A., Napoli, Italy), versus 60% of patients treated with Connettivina^®^ Bio Plus (Fidia Farmaceutici S.p.A., Abano Terme, Italy). Relying on this assumption, the data of 29 patients per group should be analyzed to obtain a power of 80% and a two-sided 5% significance level. Given an anticipated dropout rate of 5%, 60 patients should be enrolled (30 per group).

### 2.1. Treatment Plan

Sixty patients complying with the admission criteria were included in the study, and randomly assigned to receive either Connettivina^®^ Bio Plus (Fidia Farmaceutici S.p.A., Abano Terme, Italy) in the form of cream and gauze, or Fitostimoline^®^ Plus (Farmaceutici Damor S.p.A., Napoli, Italy) in the form of cream and gauze. A block randomization was generated using a computer and prepared by an investigator with no clinical involvement in the study. Informed consent was obtained from a member of the medical staff, and the physician made a phone call to an investigator who was independent of the recruitment process to assign participants to interventions.

The study plan included a six-week observation period; it was organized as follows: on the baseline visit (V1), randomization was accomplished, and after proper information, informed consent was signed. Patients assigned to Group A (Connettivina^®^ Bio Plus Fidia Farmaceutici S.p.A., Abano Terme, Italy) were treated as follows: Connettivina^®^ Bio Plus cream (Fidia Farmaceutici S.p.A., Abano Terme, Italy), one application every 24 h; Connettivina^®^ Bio Plus gauze (Fidia Farmaceutici S.p.A., Abano Terme, Italy), one application every 24 h. Patients assigned to Group B (Fitostimoline^®^ Plus) were treated as follows: Fitostimoline^®^ Plus cream (Farmaceutici Damor S.p.A., Napoli, Italy), one application every 24 h; Fitostimoline^®^ Plus gauze (Farmaceutici Damor S.p.A., Napoli, Italy), one application every 24 h. Wound dressing was performed as follows: the wound bed was uniformly covered with cream, then soaked gauzes were applied and covered with a sterile gauze; bandaging was performed if necessary. If needed, a surgical debridement was performed by the Principal Investigator (C.A.) at the clinic before the baseline visit. Follow-up visits were scheduled every 7 ± 1 days (V2, V3, V4, and V5), and the final visit (V6) was planned after 45 ± 2 days. The number of planned visits could be lower than previously stated in the case of healing or withdrawal from the study. An unplanned visit could be held if required by the patient. During V1, the informed consent was signed, and personal data (age, weight and height, medical history, vital parameters, and ongoing drug therapies) were collected; a picture of the lesion was taken. At the intermediate visits and at the last visit (V2–V6), eventual therapy changes, vital parameters, and the evaluation of eventual side effects were investigated. During every visit, a physical examination of the wound and the assessment of related symptoms were performed; moreover, the evaluation of wound edges and perilesional area was carried out. The physical exam of the wound, including location and size, the presence of fibrin, granulation tissue, infection, and maceration of the wound edges, was evaluated too. The physical exam of the wound edges (defined as the external margins of the lesion) included the assessment of erythema, bleeding, pain, burn, and itch; each of them was scored on a scale of 0 to 3 (0 = absent, 1 = mild, 2 = moderate, and 3 = severe). The physical exam of perilesional skin (the skin immediately adjacent to the wound edges) included the assessment of erythema, edema, pain, burn, itch, and dryness; the score system was the same as previously described. All the scores were summed to obtain the Total Symptoms Score (TSS); this was calculated for both the wound edges and perilesional skin. From V2 onwards, tolerability and adherence to the treatment were considered, and in the case of any systemic or local adverse event, patient withdrawal from the study was mandatory (Table 1).

The trial adhered to established procedures to maintain separation between staff that took outcome measurements and staff that delivered the intervention.

### 2.2. Endpoints

Primary endpoint: The main goal of this study was the evaluation of the efficacy of HA and silver sulfadiazine in the form of soaked gauzes and cream, compared to Rigenase^®^ and polyhexanide in the same forms. The assessment was based on the wound healing rate (WHR), evaluated as the rate of the reduction in the wound area when compared to the baseline visit (V1). Total wound healing was considered as the complete healing of the acute lesion assessed in V6 or during an earlier visit; partial wound healing was considered as incomplete healing achieved in V6.

**Figure 1:**
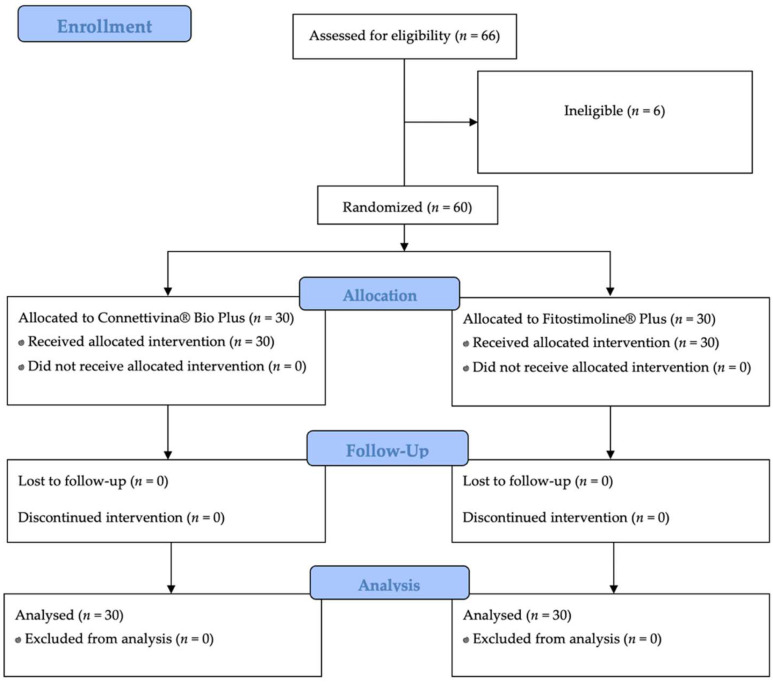
Schematic flowchart of the study.

Secondary endpoints: The evolution of the wound edges and perilesional skin was based on signs and symptoms, and these were evaluated according to the Total Symptoms Score (TSS). The tolerability of both study drugs was assessed. The schematic flowchart of the study is presented in Figure 1.

### 2.3. Statistical Analysis

Statistical analysis was performed using SPSS Statistics software package version 25 (IBM Corp. SPSS Statistics for Windows, New York, NY, USA). Parametric data were provided as mean ± standard deviation and range. The homogeneity of the study groups was evaluated using the two-tailed Mann–Whitney test, Chi-squared, Z-test, and the Kruskal–Wallis test. The primary endpoint was investigated using the Z-test and the Kruskal–Wallis test. The secondary endpoint was investigated using the two-tailed Mann–Whitney test. The significance was set at a value of *p* < 0.05.

## 3. Results

Sixty patients affected with acute superficial skin lesions of any origin were recruited and randomly assigned to a treatment group (Group A, *n* = 30; Group B, *n* = 30). Six patients were excluded due to ineligibility.

The average age of the patients was 58.5 ± 15.8 years. The average number of days elapsed between V1 and complete healing or V6 was 42.3 ± 6.2 days in Group A and 35.4 ± 8.2 days in Group B (Table 2).

**Figure 2:**
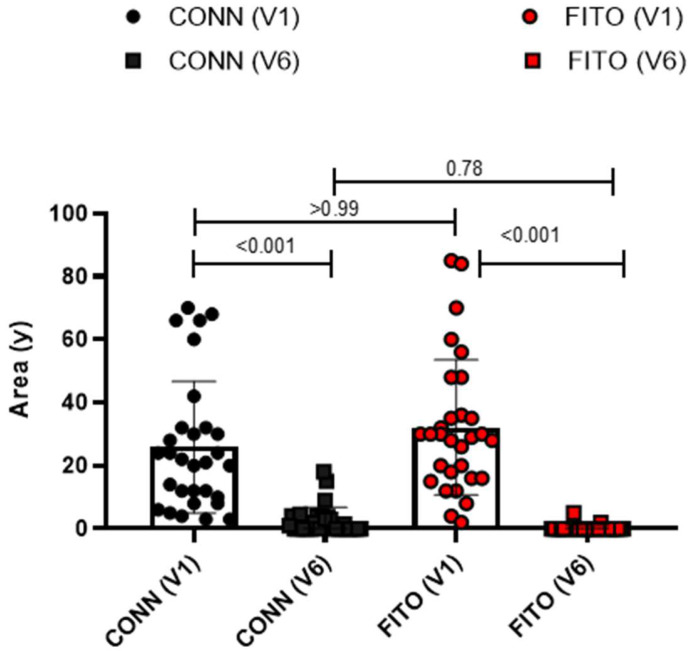
Effectiveness of treatment protocols. Conn(v1), wound area in patients treated with Connettivina^®^ Bio Plus in V1; Conn (V6), wound area in patients treated with Connettivina^®^ Bio Plus in V6; Fito(v1), wound area in patients treated with Fitostimoline^®^ Plus in V1; Fito (V6), wound area in patients treated with Fitostimoline^®^ Plus in V6.

As shown in Table 3, there were no significant differences between the two groups in terms of age, sex, area of the skin lesion assessed at V1(baseline), and wound etiology. The mean lesion area progressively decreased from baseline to V6. Both treatment protocols were effective (*p* < 0.001) (Figure 2) (Table 3).

**Figure 3:**
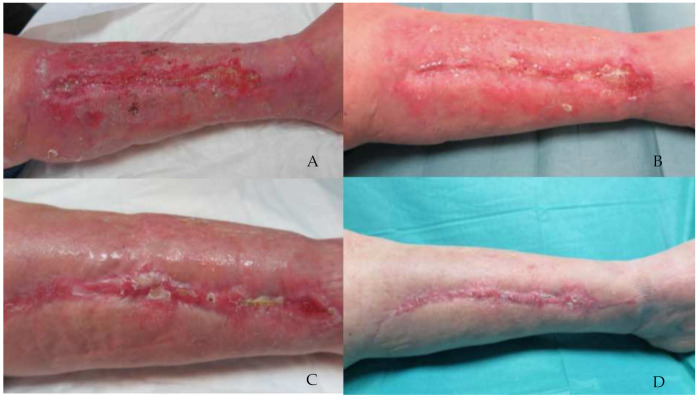
A 75-year-old female patient presented with a wound dehiscence localized on the medial aspect of the left leg. The patient was randomly assigned to the group treated with Connettivina^®^ Bio Plus cream and gauze. (**A**) Patient in V1. Wound bed is partially covered with fibrin, wound borders and perilesional skin are erythematous, and edema is observed. (**B**) Wound in V3, wound area sensibly reduced, wound bed exudate decreased considerably, although fibrin remained. Perilesional skin and wound borders improved overall. (**C**) Wound in V5. (**D**) V6, wound completely healed, and perilesional skin was a physiologic color.

**Figure 4:**
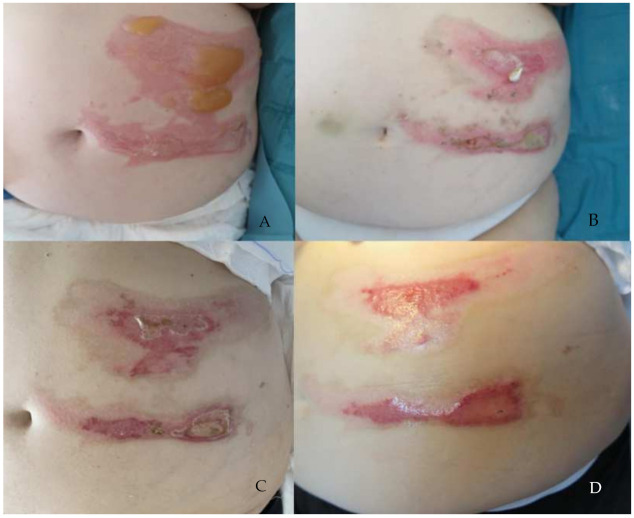
A 69-year-old female patient presented with a first- and second-degree burn lesion localized on the left side of the abdomen. The patient was randomly assigned to the group treated with Fitostimoline^®^ Plus cream and gauze. (**A**) Patient in V1, wound bed is covered with blisters; wound borders and perilesional skin are erythematous and edematous. (**B**) Patient in V3, wound area sensibly lessened (first-degree burn lesion); fibrin could be detected on the wound bed. Edema and erythema subsided significantly. (**C**) Wound in V5. (**D**) V6, wound healed completely.

Total wound healing was achieved in 17 patients undergoing treatment with Connettivina^®^ Bio Plus, and in 28 patients undergoing treatment with Fitostimoline^®^ Plus. The greater effectiveness of Fitostimoline^®^ Plus was significant (*p* = 0.001, risk ratio 0.15 (95% CI 0.04 to 0.62)). The reduction in the wound area was assessed. The wound healing rate was greater in Group B; however, these data were not statistically significant. A reduction in fibrin and maceration of the wound edges was observed in both treatment groups; however, Group B showed more satisfying results regarding reduction of fibrin on the wound bed (*p* = 0.04, risk ratio 0.2 (95% CI 0.02 to 1.70)) (Table 4) (Figure 3 and Figure 4).

Wound edges and perilesional skin TSS reduction was evaluated, and no statistical difference was observed between the study groups, *p* = 0.28 and *p* = 0.99, respectively. The TSS is a good clinical method for following the improvement related to a specific patient; however, some of the domains are merely subjective, and this could be a limitation of this evaluation method.

## 4. Discussion

Acute superficial skin lesions arising after burns, traumas, or as a complication of surgical procedures are major concerns [18,19,20,21]. These lesions could be challenging, especially in older and complex patients, if lower limbs are involved, or if an infection occurs [22,23,24]. When an acute skin injury occurs, the ECM array is altered in association with other mediators, and HA helps with maintaining the structural integrity of the skin; moreover, it creates a favorable environment for fibroblasts, which lead the way to develop proper granulating tissue [25]. HA was conjugated with SSD to create the advanced dressing known as Connettivina^®^ Bio Plus. This combination helps to prevent one of the downsides of SSD, which is delayed wound healing. Although the exact mechanism is yet to be fully understood, some data suggest that SSD impairs the cytokine milieu that results in aberrant recruitment and the activation of macrophages [26]. However, SSD retains a relevant bactericidal effect: through the impairment of DNA replication, it generates an increase in cell-wall permeability and the formation of free radicals [27].

Rigenase^®^, on the other hand, shows excellent skin repair properties. This plant-derived polysaccharide has the ability to induce the biosynthesis and release of specific proteins from keratinocytes. The majority of these secreted proteins are effectors in cell cross-talk and are involved in tissue repair and regeneration. In particular, Rigenase^®^ favors cell migration and stimulates the synthesis of new ECM [28,29,30]. The cationic polymer polyhexanide, an active factor in Fitostimoline^®^ Plus, interferes with the stability of bacterial cell membrane binding to anionic phospholipids. At the same time, its interaction with human cells is very limited, making the risk–benefit ratio superior to other antimicrobial agents [31]. Soaked gauzes and cream formulations of Connettivina^®^ Bio Plus and Fitostimoline^®^ Plus are frequently used, not only for the treatment of acute skin lesions, but also chronic wounds, burns, and pressure sores. Moreover, these are often used as dressings of skin flaps and skin grafts following reconstructive surgery [32,33,34,35] and could be used with negative pressure treatments [36,37]. Costagliola et al. [38] showed that HA formulations were effective and well tolerated for the treatment of second-degree burns. It was also demonstrated that HA-based products could enhance healing following surgery or laser skin resurfacing [14,39]. Martini et al. [40] investigated the effects of two different formulations of TVE (soaked gauzes and cream) in comparison to a gel form of equine catalase (Citrizan) for the topical treatment of small-to-medium-sized second-degree burns. The authors found that the healing rates of burn lesions and re-epithelialization >95% were higher in the Fitostimoline^®^ soaked gauzes and gel-pooled groups than in the Catalase gel group. To the best of our knowledge, the efficacy and tolerability of Connettivina^®^ Bio Plus and Fitostimoline^®^ Plus have never been compared for the treatment of acute superficial skin lesions. In the present study, every patient was randomly assigned to a treatment group. Seventeen patients in the group (A) and twenty-eight patients in the group (B) recovered completely. A greater reduction in the wound area was observed in Group B; however, these data were not statistically significant, probably due to the low numbers of the cohorts in exam; nevertheless, this is the first study comparing both medications, and there are no similar studies with a larger number of patients. Both the treatment drugs were effective in reducing the fibrin within the lesion; however, in Group B, significant major effectiveness was observed. These findings are consistent with the current literature: it is widely demonstrated that a reduction in fibrin promotes the removal of corrupted matrix and stimulates the accumulation of a competent provisional matrix, thus facilitating a physiological healing process [41,42,43]. Data available from the literature about improvement in signs and symptoms after treatment with either TVE or HA are limited. HA showed pain-relieving activity in osteoarthritis patients and for periodontitis [44]. Cellular oxidative stress plays a significant role in burn symptoms; consequently, the antioxidant activity of Fitostimoline^®^ Plus may be a key factor that either blocks or scavenges free radical generation in inflammatory tissue [45]. According to the literature, HA presents an excellent reduction in burning sensation [46]; erythema could be downgraded by both HA and TVE [47,48,49]. The TSS is a good clinical method to follow improvement related to a specific patient. However, some of the domains are merely subjective, and this could be a limitation of this evaluation method. The sample size, although conspicuous, is a limitation of the present study. The aim of our study was to assess the effectiveness of Rigenase^®^ and HA for the treatment of the most common acute superficial skin lesions, including burn, traumatic wound, or surgical wound dehiscence. The disarray of ECM and the need for a firm approach for fast recovery are major common points. We are aware that even though the pathological mechanisms in burn and surgical wounds differ [50,51], the skin biomechanics and reepithelization could be comparable, and for this reason, we chose to include all of them in our study. The frequency of each type of wound (burn, post-surgical, and traumatic) was tested to exclude any statistically significant difference between the two treatment groups. However, a prospective, multicenter study that evaluates treatment outcomes of a single specific type of wound (i.e., burn) should be advocated. The results of the present trial could help with designing future studies, and physicians that are called to manage acute skin wounds could be aided by our findings.

## 5. Conclusions

Both Connettivina^®^ Bio Plus and Fitostimoline^®^ Plus are secure and effective for the treatment of acute superficial skin lesions. Fitostimoline^®^ Plus was proven to be more effective than Connettivina^®^ Bio Plus in healing acute superficial skin lesions; moreover, it was more effective in wound healing if fibrin had been observed in the wound bed.

## Figures and Tables

**Table 1:** Study design.

	V1	V2	V3	V4	V5	V6
Informed consent	●					
Evaluation of admission criteria	●	●	●	●	●	●
Randomization	●					
Demographical data	●					
Anamnestic data	●					
Associated therapies	●	●	●	●	●	●
Vitals (BP, BPM, T) ^1^	●	●	●	●	●	●
Clinical examination of the lesion	●	●	●	●	●	●
Planimetry of lesion	●	●	●	●	●	●
Side effects		●	●	●	●	●

^1^ BP, Blood Pressure; BPM, Beats per Minute; T, Temperature.

**Table 2:** Patients and treatments.

Variable	Value	
Patients	60	
Age, years	58.5 ± 15.8	
Gender, female	28 (46.6%)	
	Group A	Group B
Days of treatment (until healing or V6)	42.3 ± 6.2	35.4 ± 8.2
Acute skin wounds		
Surgical wound	19 (63.3%)	14 (46%)
Burn	2 (6.6%)	5 (16.6%)
Trauma	9 (30%)	11 (36.6%)

Group A. Connettivina^®^ Bio Plus; Group B. Fitostimoline^®^ Plus.

**Table 3:** Differences in gender, age, wound area, and wound etiology between the populations under exam.

	Group A	Group B	*p*
Gender, m	16	15	0.79
Age, years	57.1 ± 14.1	59.9 ± 17.5	0.14
Wound area (V1), cm^2^	25.9 ± 20.8	32.1 ± 21.4	>0.99
Wound etiology			
Surgical wound	19	14	0.19
Burn	2	5	0.22
Trauma	9	11	0.58

Group A, Connettivina^®^ Bio Plus; Group B, Fitostimoline^®^ Plus.

**Table 4:** Treatment and outcomes.

Outcome	Group A	Group B	*p*-Value	Risk Ratio (95% CI)
Total wound healing, patients	56.6% (17)	93.3% (28)	0.001	0.15 (0.04 to 0.62)
Wound area, cm^2^	V1: 25.9 ± 20.8 [95% CI 18.1 to 33.6]	V1: 32.1 ± 21.4 [95% CI 24.1 to 40.1]	0.78	
	V6: 2.4 ± 4.4 [95% CI 0.8 to 4.1]	V6: 0.2 ± 1.0 [95% CI −0.13 to 0.6]		
Fibrin on wound bed, healed patients	77% (10)	95% (21)	0.04	0.2 (0.02 to 1.70)
Maceration of wound edges, healed patients	100% (6)	100% (7)	0.90	0.88 (0.02 to 38.59)

Group A, Connettivina^®^ Bio Plus; Group B, Fitostimoline^®^ Plus.

## Data Availability

The data presented in this study are available on request from the corresponding author C.A. The data are not publicly available due to privacy restrictions.
